# SDF-1α gene-activated collagen scaffold enhances provasculogenic response in a coculture of human endothelial cells with human adipose-derived stromal cells

**DOI:** 10.1007/s10856-021-06499-6

**Published:** 2021-03-06

**Authors:** Ashang L. Laiva, Fergal J. O’Brien, Michael B. Keogh

**Affiliations:** 1 Tissue Engineering Research Group, Department of Anatomy and Regenerative Medicine, Royal College of Surgeons in Ireland, 123 St. Stephen’s Green, Dublin 2, Ireland; 2 Department of Biomedical Science, Royal College of Surgeons in Ireland, Adliya, Bahrain; 3 Trinity Centre for Bioengineering, Trinity Biomedical Sciences Institute, Trinity College Dublin, Dublin 2, Ireland; 4 Advanced Materials and Bioengineering Research Centre, Royal College of Surgeons in Ireland and Trinity College Dublin, Dublin, Ireland

## Abstract

Novel biomaterials can be used to provide a better environment for cross talk between vessel forming endothelial cells and wound healing instructor stem cells for tissue regeneration. This study seeks to investigate if a collagen scaffold containing a proangiogenic gene encoding for the chemokine stromal-derived factor-1 alpha (SDF-1α GAS) could be used to enhance functional responses in a coculture of human umbilical vein endothelial cells (HUVECs) and human adipose-derived stem/stromal cells (ADSCs). Functional responses were determined by (1) monitoring the amount of junctional adhesion molecule VE-cadherin released during 14 days culture, (2) expression of provasculogenic genes on the 14th day, and (3) the bioactivity of secreted factors on neurogenic human Schwann cells. When we compared our SDF-1α GAS with a gene-free scaffold, the results showed positive proangiogenic determination characterized by a transient yet controlled release of the VE-cadherin. On the 14th day, the coculture on the SDF-1α GAS showed enhanced maturation than its gene-free equivalent through the elevation of provasculogenic genes (SDF-1α—7.4-fold, CXCR4—1.5-fold, eNOS—1.5-fold). Furthermore, we also found that the coculture on SDF-1α GAS secretes bioactive factors that significantly (*p* < 0.01) enhanced human Schwann cells’ clustering to develop toward Bünger band-like structures. Conclusively, this study reports that SDF-1α GAS could be used to produce a bioactive vascularized construct through the enhancement of the cooperative effects between endothelial cells and ADSCs.

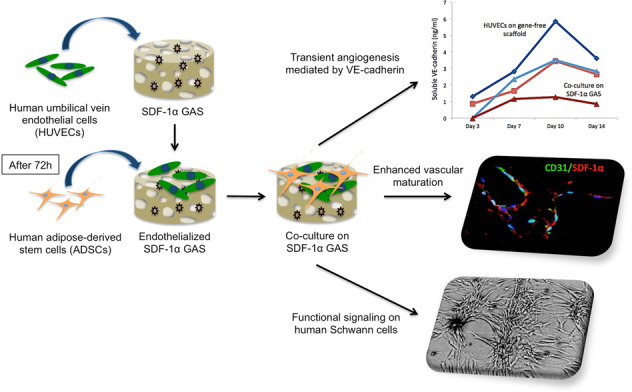

## Introduction

Insufficient regeneration of a vascular network remains one of the major limitations of biomaterials scaffolds used in wound healing [1]. To overcome this problem, functionalizing the scaffolds with either natural or synthetic proangiogenic growth factors to promote local angiogenesis is a popular strategy [2–5]. These scaffolds are designed to generate a gradient of growth factors to stimulate vessel growth toward the desired site and promote their maturation [6]. However, there is a lack of general consensus on the optimal dose of growth factors to be delivered. In addition, these angiogenic factors are susceptible to proteolysis, while bolus delivery of high doses to sustain the therapeutic effect raises the risk of toxicity as well as cost [7]. Parallel investigations such as prevascularizing biomaterial scaffolds using endothelial cells show potential [8–10]. Application of these prevascularized scaffolds is expected to significantly promote biomaterial integration as well as accelerate vascular regeneration by anastomising with host vessels [11, 12]. However, maintaining the stability of the vascular structure within the scaffold remains an important developmental criteria for therapeutic success. Consequently, strategies aiming at improving the stability of the endothelial network while allowing adequate angiogenesis continues to be investigated [13, 14].

One of the strategies adopted in our group for enhancing the stability of the endothelial network is the delayed addition of stem cells to a preformed endothelial network. This approach has also been found to enhance vascularization in vivo [10]. Having observed the importance of cross talk between endothelial cells and stem cells, we hypothesized that the endothelial cells could be activated to recruit surrounding stem cells and enhance their interaction. Biomaterial scaffolds functionalised with therapeutic genes, called the gene-activated scaffolds (GAS), are an effective platform for activating cells. It works by inducing overexpression of the therapeutic protein by the cells, eventually aiding in enhancing the regenerative capacity of the scaffold [15–17].

Our group focuses in the development of nonviral based GAS through the use of nonviral vectors such as polyethyleneimine (PEI) [16, 18, 19]. Using this vector-based GAS, we have shown that the delivery of a proangiogenic gene stromal-derived factor-1 alpha (SDF-1α) to mesenchymal stem cells (MSCs) significantly potentiates its angiogenic action on endothelial cells [20]. Moreover, SDF-1α is a potent chemokine upregulated during the early stages of wound healing. It is crucial for the homing of endothelial progenitor cells to ischemic sites and promote local angiogenesis [21–24]. Therefore, in this study, we seek to investigate if the SDF-1α GAS could be used to enhance the provasculogenic maturation of cocultures of endothelial cells and adipose-derived stem/stromal cells (ADSCs). The previous study [10] from our group used human bone marrow MSCs to reinforce the endothelial network. However, in our study, we seek to use human stem/stromal cells derived from the adipose tissue (ADSCs) because of the relative abundance of bone marrow MSCs, ease of isolation, and good patient compliance [25]. Several studies have also shown that ADSCs possess strong vasculogenic properties as well as demonstrate great potential for enhancing vascularization of various scaffolds by endothelial cells [25–29].

Nevertheless, in addition to vasculogenesis, neuronal regeneration is an important aspect of wound healing that is often overlooked. Schwann cells (SCs) are the key mediators of neurogenesis [30]. During angiogenesis, the angiogenic vessels guide the SCs to the site of wound [31]. The SCs then lay down matrix to guide regenerating axons and promote their reinnervation of the epidermis [32]. Moreover, SCs also possess the ability to provide contact guidance to endothelial cells and direct their morphogenesis toward a proangiogenic growth [33].

Therefore, the specific aims of this study are to determine if (1) the transfection of SDF-1α gene induces the secretion of SDF-1α proteins by human endothelial cells and that the protein possess chemotactic activity on human ADSCs; (2) SDF-1α GAS can support angiogenesis and promote provasculogenic response in endothelial cells and its coculture with ADSCs; and ultimately if (3) the vascularized construct can signal human SCs toward a proneurogenic response.

## Materials and methods

### Plasmid propagation and preparation of polyplex

Plasmid DNA (pDNA) encoding for the therapeutic gene SDF-1α (pSDF-1α) was obtained from InvivoGen, San Diego, USA. The plasmids were first amplified by transforming chemically competent DH5α *E. coli* cells (Biosciences, Ireland) according to the manufacturer’s protocol. Transformed cells were then expanded in lysogeny broth (LB) plates containing 100 μg/ml of blasticidin as the selective antibiotic for pSDF-1α. After 24 h at 37 °C, bacterial colonies were harvested and amplified in LB broth containing the appropriate antibiotic and cultured overnight in a shaker incubator at 37 °C. Plasmid purification was performed using a QIAGEN^®^ EndoFree^®^ Plasmid Maxi kit (Qiagen, Sussex, UK) and final nucleic acid concentration was determined using NanoDrop 1000 spectroscopy. Plasmids were further diluted in TE buffer to obtain a working concentration of 0.5 μg/μl and stored at −20 °C until use. pDNA encoding a nontherapeutic Gaussia luciferase (pLuc) purchased from New England Biolabs, Massachusetts, USA, was similarly amplified using ampicillin as the selective antibiotic. Based on our previous study, polyplex particles were formulated by initially mixing a specified amount of branched cationic 25 kDa PEI (Sigma-Aldrich, Ireland) and anionic pDNA (fixed at a dose of 2 μg) to give an N/P ratio of 10.

### Expansion and transfection of cells

Human umbilical vein endothelial cells (HUVECs) and ADSCs were obtained from Cell Applications, Inc. and iXCells Biotechnologies, USA, respectively. HUVECs were cultured in supplemented EndoGRO media (EGM-2; Merck Millipore, USA) without VEGF (EGM^−VEGF^) and expanded to passage 4 for all experiments. ADSCs were expanded in 1:1 Dulbecco’s Modified Eagles Medium/Nutrient mixture F-12 (D8437, Sigma-Aldrich, UK) supplemented with 10% FBS (Gibco, UK), 2% penicillin/streptomycin (Sigma-Aldrich, UK), and 1% amphotericin B (Gibco, UK) and harvested at passage 5 for subsequent experiments. The HUVECs were seeded at a density of 5 × 10^4^ cells per well in six-well adherent plates (Corning, Costar, UK) 24 h prior to transfection. One hour prior to transfection, the media were removed and replaced with Opti-MEM (Gibco, UK). Meanwhile, PEI was mixed with a 2 µg dose of pDNA at an N/P ratio of 10 and was allowed to assemble into polyplex by electrostatic interaction for 30 min. The PEI–pDNA polyplexes were then suspended in Opti-MEM and added to the monolayer and incubated at 37 °C for 15 min. After 15 min, an additional 1 ml of Opti-MEM was added and the cells were incubated for ~4 h. After this transfection period, the medium was removed and the cell monolayer was rinsed with PBS. EGM^−VEGF^ was then added and the cells were incubated at 37 °C to allow expression of the transgene. Media change was performed by collecting 1 ml of the media (conditioned media (CM)) and replacing it with fresh media at 3, 7, 10, and 14 days. All CM were stored at −80 °C until analysis.

### Effect of transfection on viability of cells

To determine the effect of therapeutic gene transfer on the transfected cell, cell viability following transfection with PEI–pSDF-1α or PEI–pLuc was assessed using the colorimetric MTS assay (CellTiter 96^®^ AQ_ueous_ One Solution, Promega, Madison, WI, USA). Briefly at 1, 3, and 7 days post transfection, 20 μl of the MTS reagent was added to the cells in 100 μl of media, and incubated for 4 h at 37 °C. Intensity of the resulting color was measured at an absorbance of 490 nm using a Multiskan GO plate reader (Thermo Scientific, UK). Cell viability percentage (%) was determined according to the equation (absorbance_[transfected]_/absorbance_[control]_) × 100, keeping the untransfected cells as 100% viability control.

### ELISA for quantification of SDF-1α protein production post transfection in 2D culture

In order to determine successful transfection of pSDF-1α, secretion of SDF-1α proteins into the culture medium was determined using a human SDF-1α specific ELISA kit (DY350, R&D Systems, UK) according to the manufacturer’s instructions. One hundred microliters of CM collected from days 3, 7, 10, and 14 were used for the assay and the absorbance was read at 450 nm using a Multiskan GO plate reader (Thermo Scientific, UK) whereby the quantity of SDF-1α protein present was deduced by calculating against a standard curve.

### Coculture of HUVECs and ADSCs in a 2D setup

In order to assess the functionality of secreted proteins, a delayed addition approach previously reported by our group [10] was adopted where stem cells are added onto preseeded HUVECs. Based on the previous study [10], the ratio of HUVECs to the ADSCs was set at 4:1. The transfection experiment was performed as in the section “Expansion and transfection of cells,” except that HUVECs were initially seeded at a density of 4 × 10^4^ and allowed to grow until the 3rd day, after which 1 × 10^4^ ADSCs were added. On the 14th day of incubation, images of the cocultures were captured using a Celestron^®^ digital microscope imager, attached to a phase-contrast Olympus CKX31 microscope. CM was also collected from these groups as described in the section “Expansion and transfection of cells,” to analyze how the expression of SDF-1α is affected by the addition of ADSCs. All the cocultures were maintained in EGM^−VEGF^.

### Cell seeding on SDF-1α GAS

SDF-1α GAS was developed as described earlier [20], which basically involves soak-loading of the PEI–pSDF-1α polyplex nanoparticles into a freeze-dried porous 3D coll-CS scaffold [34]. For this study, a double-sided cell seeding of equal cellular density was performed. For culture of HUVECs alone, a total of 5 × 10^5^ cells were seeded onto the scaffold/SDF-1α GAS, while for coculture groups, 4 × 10^5^ HUVECs were initially seeded and allowed to endothelialize the scaffolds/SDF-1α GAS for 3 days before 1 × 10^5^ ADSCs were added onto it [10]. Consequently, four groups were developed (1) HUVECs alone on gene-free scaffold, (2) HUVECs alone on SDF-1α GAS, (3) coculture on gene-free scaffold, and (4) coculture on SDF-1α GAS. For initial transfection of HUVECs in the SDF-1α GAS, 2 ml of Opti-MEM was added and the plates were incubated at 37 °C for 24 h after which the media were removed and replaced with 2 ml of EGM^−VEGF^. To assay the temporal regulation of soluble vascular molecules, media were collected and replaced in the same manner as described previously in the section “Expansion and transfection of cells.”

#### ELISA for quantification of soluble vascular endothelial cadherin (VE-cadherin) and VCAM-1 in endothelialized SDF-1α GAS

To determine angiogenic growth of the endothelial cultures in SDF-1α GAS, secretion of the soluble forms of endothelial junctional adhesion molecule VE-cadherin (DY938, R&D Systems, UK) and inflammation induced transmembrane molecule VCAM-1 (DY809, R&D Systems, UK) was analyzed. To assay angiogenesis, 100 μl of CM collected from days 3, 7, 10, and 14 were used for the assay and the absorbance was read at 450 nm using a Multiskan GO plate reader (Thermo Scientific, UK). The quantity of soluble VE-cadherin or VCAM-1 released was deduced by calculating against their respective standard curves.

#### qRT-PCR analysis to determine functional gene expression in endothelialized SDF-1α GAS

Cells from the scaffolds were harvested on the 14th day for gene expression analysis. RNA extraction and reverse transcription was performed as described in previous chapter. qRT-PCR was then performed on cDNA using the following primers Hs_CXCL12_1_SG, Hs_CXCR4_1_SG, and Hs_NOS3_1_SG, which encodes for human SDF-1α, CXCR4, and eNOS, respectively. Fold change in mRNA expression relative to the untransfected control was calculated using the 2^−∆∆^CT method [35] from averages of three samples for each group. GAPDH (Hs_GAPDH_1_SG) was used as the housekeeping gene.

#### Immunofluorescent imaging

After 14 days of culture, samples were processed for immunofluorescent imaging. Briefly, the samples were first washed with PBS and fixed in 10% neutral buffered formalin for 20 min, and then processed using the standard protocol for paraffinization. The samples were then cut into 8-μm thick slices, deparaffinized, and mounted on slides. Prior to staining, the cells were permeabilized with 0.2% Tween^®^ 20 (Sigma-Aldrich, France) solution in 1x PBS for 30 min (10 min wash × 3) and blocked using 10% normal goat serum (Invitrogen, UK)/5% BSA/0.3 M glycine (prepared in permeabilizing solution) for 1 h to inhibit nonspecific protein interaction. The slides were then stained using monoclonal antibodies of CD31 (1:50; ab119339, Abcam, UK) and SDF-1α (1:100; ab155090, Abcam, UK) to visualize endothelial morphogenesis and compare the expression of SDF-1α proteins, respectively.

The next day, the slides were rinsed in PBS thrice for 2–3 min each to remove any unbound primary antibodies. Subsequently, the slides were incubated in either Alexa 488-conjugated goat anti-mouse IgG (A32723, Invitrogen, UK) or Alexa 594-conjugated goat anti-rabbit IgG (A11012, Invitrogen, UK) at 1:800 dilution at room temperature for 1 h in dark. The rinsing step was performed as before and stained for nuclei using the mounting medium with DAPI (ab104139, Abcam, UK) and covered with cover slips. Images were then captured using fluorescence microscope (Olympus BX43, Japan) at 40× magnification. Samples incubated with only secondary antibodies were used as controls. All the antibodies were diluted in 1% BSA in PBS prior to use.

#### Image analysis

The “ImageJ” software (ImageJ, NIH, Maryland, USA) was used to semiquantitatively determine the amount of expressed proteins. For each marker, a constant threshold value was first determined through preliminary imaging of various sections. Using the set threshold value, integrated density (stained area × mean gray value) of the images was determined and then normalized to the number of cells (DAPI counting) to give a final mean fluorescence density per cell. An average was quantified from four to five random images per sample, with a minimum of three samples per group. The averages obtained from each group were then used for statistical comparisons between the groups.

#### Bioactivity analysis of secreted factors from coculture on SDF-1α GAS on human SCs

In order to study if the vascularized construct possesses proneurogenic properties, CM (day 7) collected from the coculture groups was exposed to human SCs (iXCells Biotechnologies, USA). Human SCs at passages 3 and 4 were seeded onto poly-l-lysine (P4707, Sigma, UK) coated 12-well plates at a density of 30,000 cells/well and allowed to grow until confluency. Injury was made to the monolayer of SCs by creating a scratch using a sterile 200 μl pipette tip. The cells were gently rinsed with PBS to remove any cellular debris and treated with CM from the vascularized constructs. Cellular organization was then observed until 48 h. Quantification of the cellular area was performed using ImageJ from at least five images per well (*n* = 3).

### Statistical analysis

All results are expressed as mean ± standard deviation. Unpaired, two-tailed, *t*-test was used to demonstrate the statistical significance between groups, where *p* < 0.05 was considered to be significant. One-way ANOVA was also used to compare statistical difference between the multiple groups, considering *p* < 0.05 to be significant.

## Results

### PEI–pSDF-1α polyplex effectively transfected HUVECs and induced the secretion of functional SDF-1α proteins

**Fig. 1:**
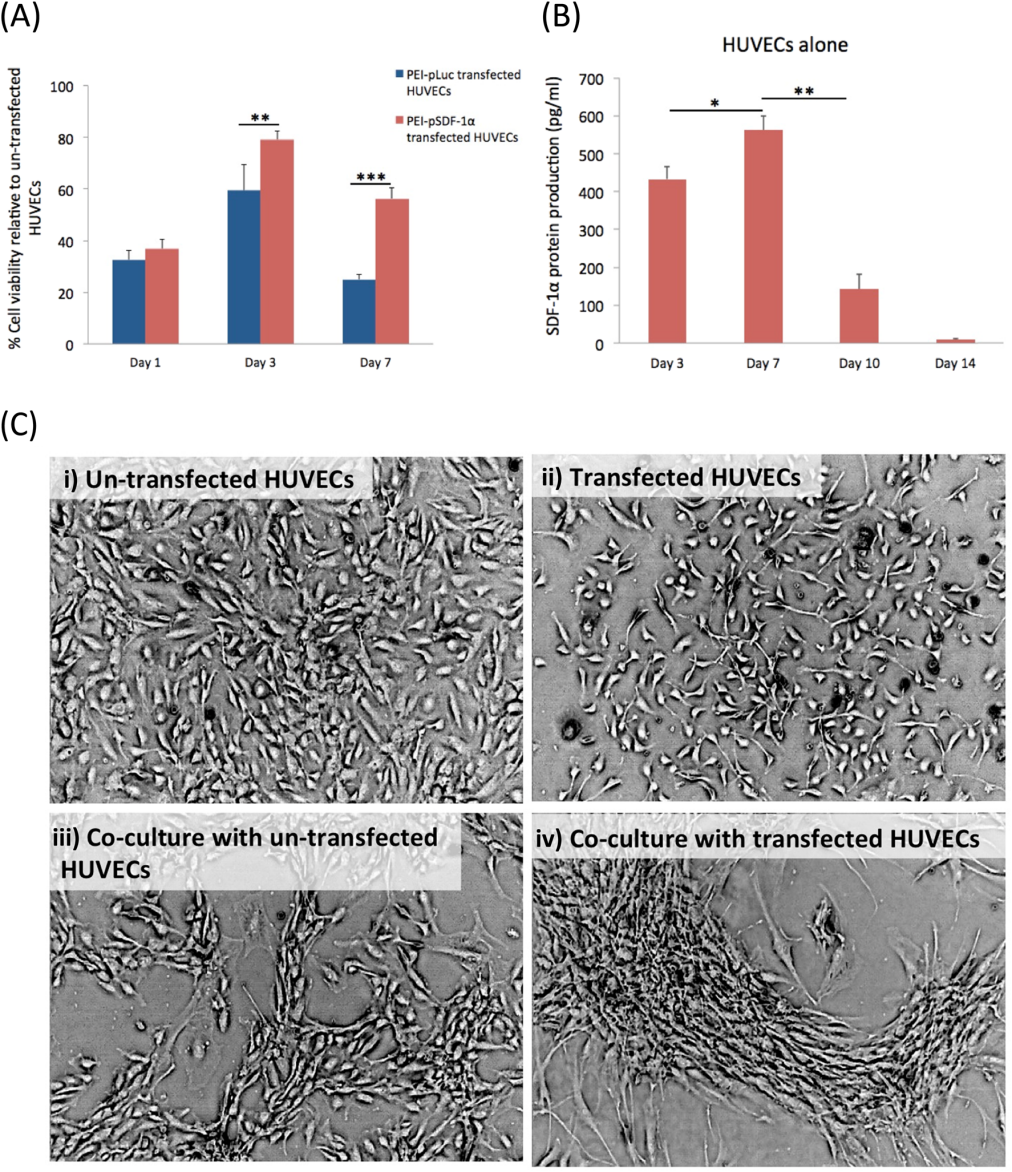
Effect of PEI–pSDF-1α transfection on HUVECs and its impact on ADSCs. **A** PEI–pSDF-1α transfected HUVECs exhibited significantly enhanced survivability response compared to those transfected nontherapeutic PEI–pLuc polyplex. **B** Transfection with PEI–pSDF-1α induced transient production of the target protein over a period of 2 weeks. **C** Phase-contrast microscopy images of morphological changes in HUVECs and its coculture with ADSCs at day 14. i Untransfected HUVECs maintained its cobblestone-like morphology. ii Transfected HUVECs appeared more polarized. iii Addition of ADSCs to untransfected HUVECs resulted in the formation of interconnected cellular network within the monolayer assembly. iv Elongated, pseudo three-dimensional dense cellular clusters were formed in transfected coculture group. Data are plotted as mean ± standard deviation (*n* = 6)

MTS assay showed that at days 3 and 7, HUVECs transfected with PEI–pSDF-1α significantly promoted their survivability than the groups transfected with polyplex carrying the nontherapeutic gene, pLuc (Fig. 1A). Subsequent SDF-1α ELISA analysis showed that protein production reached the maximum (565 ± 35 pg/ml) on the 7th day but dropped significantly (*p* < 0.001) at later time points (Fig. 1B). Prior to the addition of ADSCs, HUVECs secreted comparable levels of SDF-1α (530 ± 95 pg/ml) as HUVECs monoculture group. However, SDF-1α protein levels in the CM were not detectable on the days after the HUVECs were cocultured with ADSCs. Furthermore, on the 14th day, phase-contrast microscopy revealed that the transfected HUVECs when cocultured with ADSCs displayed the formation of elongated cellular clusters with cellular extensions appearing out of the cluster in multiple directions (Fig. 1C-iv). The untransfected coculture group also exhibited the formation of interconnected cellular network. However, cobblestone-like morphological features of the individual endothelial cells remained distinguishable (Fig. 1C-iii). Clearly, morphological differences between the coculture groups suggest that PEI–pSDF-1α transfected HUVECs secrete functional proteins capable of recruiting ADSCs for enhanced functional interaction.

### SDF-1α GAS effectively engages endothelial VE-cadherin while promoting angiogenesis

**Fig. 2:**
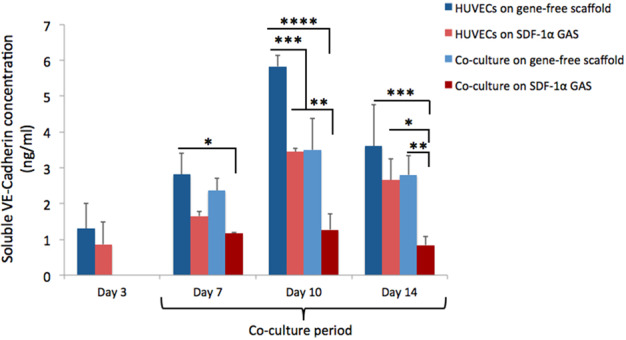
Temporal regulation of soluble VE-cadherin from endothelialized gene-free scaffold and SDF-1α GAS. SDF-1α GAS strongly affects the vascular growth of endothelial cells by suppressing the release of soluble VE-cadherin. Coculturing with ADSCs offers further control on the release of soluble VE-cadherin from endothelial cells. HUVECs on SDF-1α GAS demonstrated significant reduction in the levels of soluble VE-cadherin at days 7 (*p* < 0.05) and 10 (*p* < 0.0005) relative to HUVECs on gene-free scaffold. Coculture on SDF-1α GAS strongly attenuated the release of VE-cadherin at all time points. Data are presented as mean ± standard deviation. One-way ANOVA was used to deduce statistical significance. *, **, ***, and **** indicate statistical significance of *p* < 0.05, *p* < 0.01, *p* < 0.005, and *p* < 0.0005, respectively

Angiogenesis is a dynamic process that occurs through transient disruption of the endothelial adherent junctions [36]. During the junctional disruption, the adhesion molecules, primarily the VE-cadherin is released as a soluble form [37]. Therefore, in order to measure angiogenesis within the SDF-1α GAS, we assayed soluble VE-cadherin released during the culture period (Fig. 2). All the groups demonstrated a transient trend in the release of soluble VE-cadherin that peaked at day 10. Among the groups, HUVECs on gene-free scaffold released the highest amount of VE-cadherin at all time points (up to 5.83 ± 0.3 ng/ml at day 10). When ADSCs were added to the preformed endothelial network, the release of soluble VE-cadherin was strongly attenuated. However, we noted a similar amount of VE-cadherin released by the HUVECs on SDF-1α GAS as that of the coculture on the gene-free scaffold. Considering the impact of SDF-1α GAS on HUVECs, we anticipated more pronounced attenuation of VE-cadherin release in the coculture on SDF-1α GAS. We confirmed this event by measuring the level of VE-cadherin from the coculture on SDF-1α GAS, which showed the lowest (1.27 ± 0.45 ng/ml at day 10) at all time points. Taken together, it implies that SDF-1α GAS engages the endothelial VE-cadherin to modulate angiogenesis in a transient manner.

**Fig. 3:**
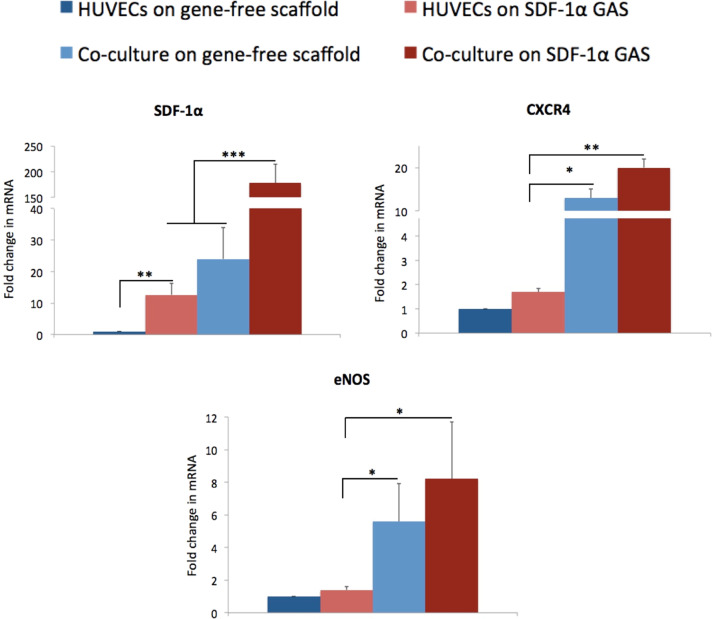
Gene expression analysis of HUVECs and its coculture on gene-free scaffolds and SDF-1α GAS. SDF-1α GAS significantly increased the expression of mRNAs for SDF-1α and its cognate receptor CXCR4 in HUVECs. Coculture with ADSCs significantly elevated the expression of downstream effector genes of SDF-1α—CXCR4 and eNOS than the HUVECs on SDF-1α GAS. Data are plotted as mean ± standard deviation (*n* = 3). *, **, and *** indicate statistical significance of *p* < 0.05, *p* < 0.01, and *p* < 0.005, respectively

The release of soluble VE-cadherin could also be mediated by inflammation and its level corresponds to the degree of inflammation [38, 39]. Therefore, having observed high level of soluble VE-cadherin with HUVECs on gene-free scaffold, we assessed if vascular inflammation was involved. This was conducted by measuring the release of inflammation-associated soluble VCAM-1 [40]. In all the groups, no detectable amounts of soluble VCAM-1 were released, supporting the notion that the release of VE-cadherin was a physiological process to promote angiogenesis.

### Coculture enhances SDF-1α-mediated provasculogenic gene expression

Having noted that cultures on SDF-1α GAS effectively preserve endothelial junctional integrity during angiogenesis, gene expression was performed at day 14 to determine endothelial maturation (Fig. 3). First, we measured the levels of SDF-1α mRNA and noted that coculturing with ADSCs enhances the expression of SDF-1α mRNA than HUVECs alone. The coculture on the gene-free scaffold expressed the SDF-1α mRNA twice as high as HUVECs on the SDF-1α GAS. A more pronounced expression was noted in the coculture on the SDF-1α GAS that showed an eightfold higher expression of the SDF-1α mRNA than its gene-free equivalent.

Provasculogenic maturation was then determined based on the expression of SDF-1α’s downstream effector mRNAs, namely CXCR4 and eNOS. We noted that the SDF-1α GAS is not sufficiently potent to drive HUVECs toward a provasculogenic maturation. The SDF-1α GAS caused a modest increase (43%) in the expression of eNOS in HUVECs. However, coculturing with ADSCs significantly upregulated the expression of eNOS than the HUVECs on SDF-1α GAS. Furthermore, the coculture on SDF-1α GAS exhibited a sixfold higher expression of eNOS mRNA than the HUVECs on the SDF-1α GAS. This finding suggests that coculture is crucial for enhancing endothelial maturation in SDF-1α GAS.

### SDF-1α GAS supports endothelial morphogenesis

**Fig. 4:**
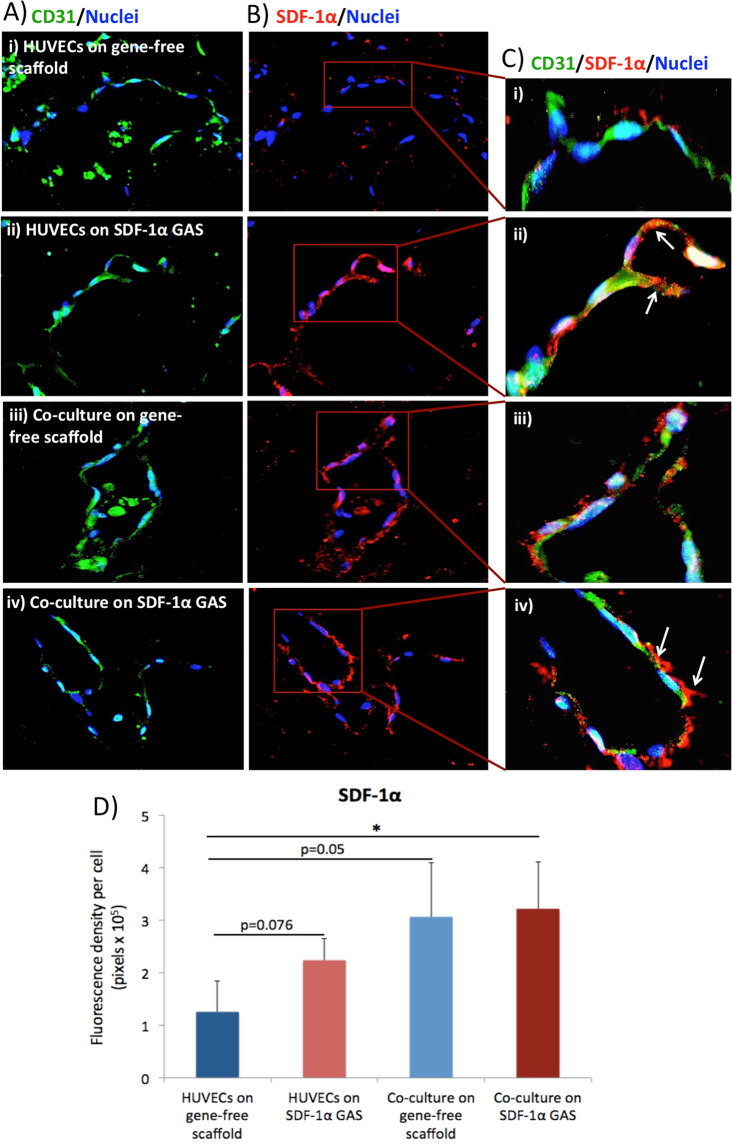
Visualization of endothelial anastomosis and SDF-1α expression. **A** All the cultures showed strong immunoreactivity to CD31 and exhibited endothelial morphogenesis representative of capillary-like network. **B** HUVECs on the gene-free scaffold expressed the lowest amount of SDF-1α proteins. **C** Magnified images of the endothelial network showing the differences in spatial distribution of SDF-1α between the groups. **D** Quantitative representation of SDF-1α protein expression showing an increasing trend toward the coculture group. Data are presented as mean ± standard deviation. * indicates statistical significance of *p* < 0.05

Immunostaining with the primary marker for endothelial cells CD31 revealed that all the groups had undergone morphogenesis representative of a capillary-like network (Fig. 4A). Having confirmed this, SDF-1α was double immunostained with CD31 (Fig. 4B, C) to visualize its spatial distribution on the cellular surface as well as the level of expression. Mean fluorescence density analysis of SDF-1α revealed a progressive increase in the expression of SDF-1α from the monocultures of HUVECs toward the coculture group (Fig. 4D). However, no significant difference was noted between HUVECs on the SDF-1α GAS and the coculture groups. Only the coculture on SDF-1α GAS exhibited significantly higher levels of SDF-1α than the HUVECs on the gene-free scaffold. Spatially, the SDF-1α in HUVECs on SDF-1α GAS appears to be bound to the cellular surface (indicated by arrows, Fig. 4C-ii). On the other hand, SDF-1α in coculture on SDF-1α GAS appears to be secreted into the extracellular space (indicated by arrows, Fig. 4C-iv). Overall, this finding demonstrates that SDF-1α GAS effectively supports angiogenesis and that coculture enhances SDF-1α protein expression.

### Coculture on SDF-1α GAS induces human SCs morphogenesis

In addition to vasculogenesis, neuronal regeneration is an important aspect of wound healing that is often overlooked. During angiogenesis, the angiogenic vessels guide the SCs to the site of wound [31]. The SCs then lay down matrix to guide regenerating axons and promote their reinnervation of the epidermis [32]. Therefore, having observed enhanced vasculogenic response in the coculture groups, the potential of the vascularized constructs to signal SCs was investigated.

**Fig. 5:**
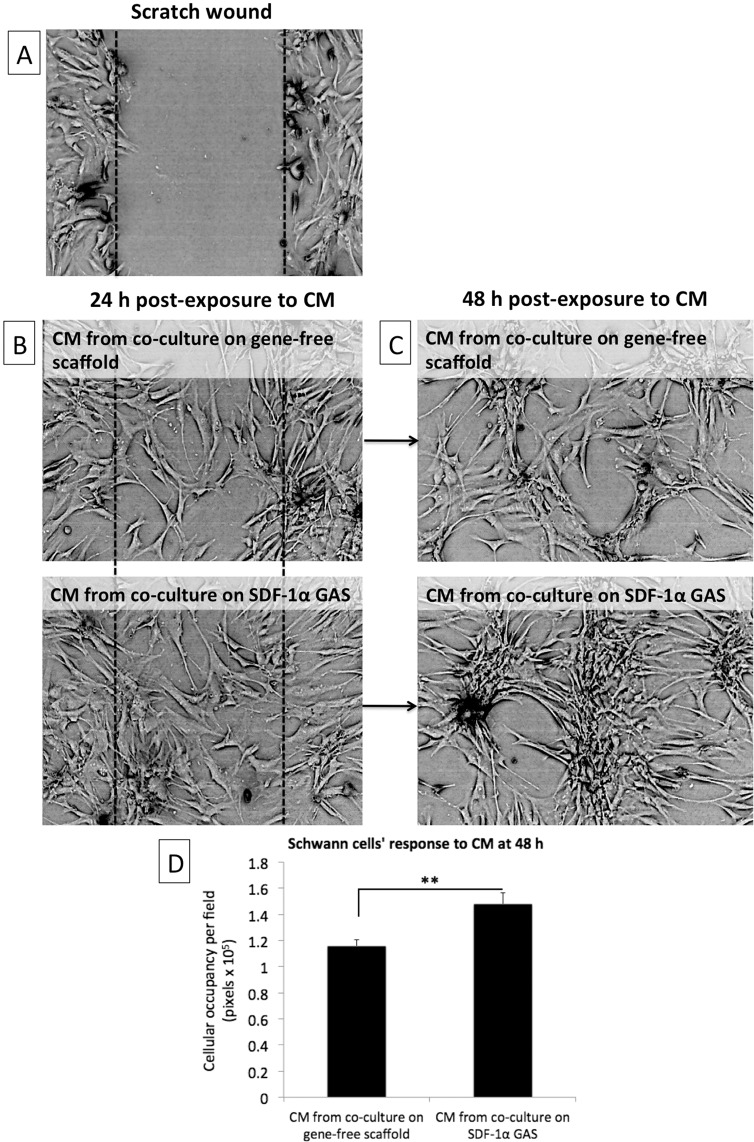
Cellular organization of human Schwann cells in response to CM derived from coculture of endothelial cells and ADSCs. **A** A scratched monolayer of Schwann cells. **B** Schwann cells invading the wounded zone 24 h post exposure to CM. **C** Schwann cells undergoing morphological changes 48 h post exposure to CM. **D** At 48 h, Schwann cells exposed to CM from the coculture on SDF-1α GAS organized into significantly (*p* < 0.01) larger interconnected clusters than the Schwann cells exposed to CM from the coculture on gene-free scaffold. Data are presented as mean ± standard deviation

Twenty-four hours post exposure to CM, the SCs actively invaded the wound zone regardless of the source of the CM (Fig. 5B). However, further incubation of the SCs in the CM up to 48 h resulted in the generation of cluster-like structures (Fig. 5C). These clusters formed connections between other clusters through slender cord-like structures that protruded out of each clusters. The clusters did not have defined boundaries however measurement of the total spatial coverage revealed that the CM from coculture on SDF-1α GAS generated significantly larger SCs clusters than the CM from its gene-free equivalent (Fig. 5C-ii). In addition, the cord-like structures connecting the large clusters were highly aligned and more parallelly arranged than the connections between the smaller clusters (Fig. 5C-ii). From these observations, it is evident that vascularized SDF-1α GAS produces signaling components that can stimulate proneurogenic behavior in SCs implicated for neuronal regeneration.

## Discussion

The overall objective of the study was to assess the bioactivity of SDF-1α GAS on human endothelial cells and its coculture with human ADSCs. A preliminary 2D study showed that the HUVECs transfected with PEI–pSDF-1α polyplex transiently secreted SDF-1α proteins that actively recruited ADSCs and enhanced their adhesion to the HUVECs. On the SDF-1α GAS, the HUVECs displayed angiogenesis that occurred in a transient manner through regulation of the junctional adhesion molecule VE-cadherin. Coculturing with ADSCs significantly improved the preservation of the VE-cadherin and also enhanced the maturation of endothelial cells through the expression of downstream effectors CXCR4 and eNOS. Secreted factors from the provasculogenic vessels on SDF-1α GAS further displayed strong bioactive properties toward human SCs that organized into Bünger band-like structures. Together, this study showed that SDF-1α GAS is a highly functional biomaterial scaffold capable of enhancing provasculogenic response for wound healing applications.

In the application of synthetic gene-delivery vectors, the survivability of the cells post transfection is critical for offering the optimal therapeutic response [41–43]. Therefore, we first evaluated the safety of the polyplexes on the HUVECs. First, we noted a proliferation curve that peaked at day 3. This appears to be a general manner of proliferation of HUVECs in EGM, as a similar case has been observed in other studies, which validated the behavior through the measurement of proliferation marker Ki67 [44]. Nevertheless, our finding indicates that despite an initial insult to HUVECs’ health due to the exposure to polyplexes, the delivery of SDF-1α gene significantly enhanced the survivability of HUVECs than the ones transfected with Luc gene (Fig. 1A). This finding possibly suggests that secreted SDF-1α proteins may have exerted a protective effect on the HUVECs itself, mediated by a positive autocrine loop [45]. Our analysis of SDF-1α protein production further proved that the transfected HUVECs indeed produced high levels of SDF-1α protein that lasted up to 14 days (Fig. 1B). We further confirmed that SDF-1α protein was also highly bioactive toward the ADSCs as a greater clustering of the cells occurred in the coculture group consisting of SDF-1α producing HUVECs. The response to SDF-1α is potentially mediated by CXCR4 in ADSCs that play a specific role in SDF-1α driven chemotaxis [46].

Normally, to measure angiogenesis on 3D scaffolds over time, the endothelialized constructs are harvested at prespecified time points and imaged for capillary-like network formation [14, 25]. Here, we show a relatively simpler alternative method that allows continuous monitoring of the endothelial growth in a 3D scaffold without the need for harvesting the cellularized samples. This technique also offers another advantage by providing information of the dynamic changes in endothelial junctional stability, which is crucial for endothelial homeostasis [36, 47]. Based on a previous study [37], which described that the endothelial junctional adhesion molecule VE-cadherin is proteolytically cleaved during angiogenesis and shed as a soluble form, we measured the amount of VE-cadherin released from the endothelialized constructs. The HUVECs on the gene-free scaffold showed a clear transient release of the soluble VE-cadherin that peaked at day 10. This finding confirms that the VE-cadherin is temporally involved in endothelial angiogenesis. Furthermore, the pattern of VE-cadherin release is also in agreement with findings of both in vitro [14, 25] and in vivo [48] studies, which showed that the angiogenesis peaks between days 7 and 14. Moreover, comparing the levels of VE-cadherin revealed a potential role of SDF-1α GAS that it may be involved in the enhancement of homophilic endothelial-to-endothelial adhesion mediated by VE-cadherin [49]. This speculation is derived from the finding that the HUVECs on SDF-1α GAS released similar levels of VE-cadherin as that of the coculture on the gene-free scaffold. The cocultures are generally known to enhance the expression of cellular VE-cadherin and engage them in the restoration of endothelial barrier functions [50–52].

The significant activation of CXCR4 in the HUVECs on SDF-1α GAS presents a potential mechanism that may have controlled the release of VE-cadherin from the HUVECs. The SDF-1α-induced CXCR4 may sustain the expression of VE-cadherin and maintain the endothelial barrier functions via the activation of Wnt/β-catenin pathway [53]. The stem cells are also known to employ the β-catenin pathway to enhance the VE-cadherin-mediated barrier properties of endothelial cells [51]. Importantly, gene expression analysis further showed that the SDF-1α GAS alone is insufficient to drive provasculogenic response in HUVECs and that coculturing with ADSCs was crucial for enhancement of the provasculogenic gene eNOS. Similar enhancement in the expression of eNOS mRNA in the coculture of endothelial cells and ADSCs has also been observed in other studies [54]. Although we investigated this study as an approach for enhancing blood vessel regeneration in a biomaterial scaffold, SDF-1α GAS may also be useful for promoting lymphangiogenesis. The lymphatic vessels regulate immune cell trafficking and tissue fluid homeostasis [55]. In addition to angiogenesis, lymphangiogenesis also actively participates in the wound healing process and relies on the growth of lymphatic endothelial cells (LECs) [56]. VEGF-C is a potent stimulant of LECs [56]. However, similar to angiogenesis, studies have found that the interaction of LECs with stem cells is crucial for expanding the lymphatic endothelial network [57, 58]. Moreover, the ADSCs, despite the low production of VEGF-C, were found to enhance the growth of LECs effectively [59]. Therefore, given the importance of endothelial cells’ interaction with stem cells for lymphangiogenesis, the ability of SDF-1α GAS to induce the overexpression of chemotactic SDF-1α may facilitate improved intercellular interaction and enhance functional growth.

In order to readdress the importance of neuronal activation while considering strategies for wound healing, we ultimately investigated if the provasculogenic coculture constructs can signal SCs. SCs were specifically chosen because, in the process of reinnervation, SCs invasion precedes axonal regeneration [60, 61]. During angiogenesis, invading blood vessels guide the SCs into the wound site [31, 61]. The SCs then lay down matrix and guide the regenerating axons for epidermal reinnervation [61].

We validated that the CM produced by the coculture on the SDF-1α GAS possessed superior bioactive properties than its gene-free scaffold equivalent. However, an apparent difference in the response of SCs appeared only at 48 h post exposure to CM. The SCs exposed to CM from the SDF-1α GAS group grew into significantly large clusters. Clusters incubated in the CM from the SDF-1α GAS group also developed parallelly arranged cord-like structures connecting other clusters. This cellular arrangement has also been demonstrated by SCs when they were cocultured with neural cells such as the meningeal cells [62, 63]. These cellular arrangement are presumed a transformation of the SCs into Bünger bands, which is necessary for supporting axonal regeneration across wound [32, 62].

To this end, we show that the provasculogenic structures created on SDF-1α GAS are highly potent bioactive constructs for wound healing applications. Nevertheless, it would be interesting to see how the SDF-1α GAS might work on adult endothelial cells such as the peripheral blood or umbilical cord blood-derived endothelial cells (ECFCs) [64] and the induced pluripotent stem cells derived endothelial cells (iPSCs-ECs) [65]. Both ECFCs and iPSCs are known to show high proangiogenic activity both in vitro [66] and in vivo [64, 65]. Moreover, ECFCs are also known to possess stem cell-like properties [67], which may offer enhanced therapeutic effects. These cell types are of particular interest as they can be harvested from the patient and representing clinically translatable cell candidates for autologous therapy [68, 69]. In the future, we will further attempt to investigate the efficacy of the prevascularized construct in vivo, which is currently a limitation of the study, using established techniques such as subcutaneous implantation in nude mice.

## Conclusion

This study explored the functional impact of SDF-1α GAS on human endothelial cells and its coculture with human ADSCs. Particularly, we have shown that SDF-1α GAS effectively supports endothelial angiogenesis by desirably controlling the junctional adhesive effect, which is essential for maturation of the endothelial network. Better maturation could be achieved through coculturing with ADSCs, which was further enhanced when supported with SDF-1α GAS. In addition, SDF-1α GAS activated coculture group demonstrated a strong capacity to signal SCs to differentiate toward a proneurogenic phenotype by influencing their cellular organization into Bünger band-like structures. Taken together, we showed a potent bioinstructive role of SDF-1α GAS for the generation of bioactive provasculogenic constructs for enhanced wound healing applications.
